# The complete chloroplast genome of a distinctive meadow-rue, *Thalictrum foeniculaceum* (Ranunculaceae)

**DOI:** 10.1080/23802359.2020.1870880

**Published:** 2021-02-08

**Authors:** Lele Lin, Jian He, Rudan Lyu, Min Yao, Yike Luo, Lei Xie

**Affiliations:** School of Ecology and Nature Conservation, Beijing Forestry University, 35 East Qinghua Rd., Haidian District, Beijing, PR China

**Keywords:** Complete chloroplast genome, phylogeny, Ranunculaceae, *Thalictrum*

## Abstract

*Thalictrum foeniculaceum* is a morphologically distinctive species in the genus with very reduced filiform compound leaves and big pinkish flowers occurring in dry slopes of northern China. Herein, we report the first complete chloroplast genome sequence of *T. foeniculaceum*. The chloroplast genome sequence was 155,923 bp in length, with large and small single-copy regions (LSC with 85,323 bp and SSC with 17,628 bp in length) separated by two inverted repeat regions (IRs with 26,486 bp). The total GC content was 38.3%. The complete plastome sequence contained 111 genes, including 77 protein-coding, 30 *tRNA*, and four *rRNA* genes. The phylogenetic analysis of *Thalictrum* based on the complete chloroplast genomes available online was also presented in this study.

*Thalictrum* is a large genus with about 200 species almost worldwide in the buttercup family (Ranunculaceae) (Tamura 1995). *Thalictrum* species are known as the Traditional Chinese Medicines (TCM): horsetail goldthread. *Thalictrum* species have monochlamydeous flower which are often not showy. However, several Chinese species have large pinkish or purple flowers, such as *T. delavayi*, *T. grandiflorum*, *T. diffusiflorum*, *T. chelidonii*, and *T. foeniculaceum* (Wang and Wang 1979) and showed horticultural value. Among them, *T. foeniculaceum* is the most distinctive one with linear leaflets presumably adaptive to dry environments. In order to better understand this species, we reported and characterized the first complete chloroplast genome of *T. foeniculaceum* using the next-generation sequencing method.

Fresh young leaves of *T. foeniculaceum* were collected from Yunju Temple, Fangshan Dist., Beijing (115.766°E, 39.609°N). Voucher specimens were deposited in the Herbarium of Beijing Forestry University (BJFC) (under collection numbers of *L. Xie 202007001*). Genomic DNAs were extracted using a genomic DNA extraction kit (Tiangen Biotech Co. Ltd., Beijing, China), and 2 × 150 bp pair-end sequencing was performed on an Illumina HiSeq 4000 platform at Novogene (http://www.novogene.com, China). We used Map function of Geneious version R11 (Biomatters, Ltd., Auckland, New Zealand, Kearse et al. 2012) to select chloroplast reads using published plastome sequence of *Thalictrum* as reference (He et al. 2019). Then, these chloroplast reads were *de novo* assembled with Geneious R11. Gaps were filled using Fine Tuning function of Geneious R11. The assembled chloroplast sequence was annotated using the Plastid Genome Annotator (PGA, Qu et al. 2019), and verified by Geneious R11.

The complete chloroplast genome sequence of *T. foeniculaceum* is 155,923 bp in length, with a large single-copy (LSC) region of 85,323 bp, a small single-copy (SSC) region of 17,628 bp, and two inverted repeats (IR) of 26,486 bp. The plastome contains 111 functional genes, including 77 protein-coding genes, 30 *tRNA* genes, and four *rRNA* genes. The total sequence GC content is 38.3%. Structural variations, such as gene inversions, transpositions, and IR expansion of this plastome sequence was consistent with other *Thalictrum* samples reported by previous studies (He et al. 2019; Zhai et al. 2019).

Complete chloroplast genome sequences of the other *Thalictrum* species available from GenBank were retrieved for phylogenetic analysis. Bayesian inference was used for phylogeny reconstruction (Figure 1). The sequence alignment and all the settings of Bayesian analyses were followed (Liu et al. 2018). Phylogenetic framework of *Thalictrum* as well as outgroups were consistent with previous molecular studies (Soza et al. 2012; He et al. 2019; Zhai et al. 2019).

**Figure 1.**
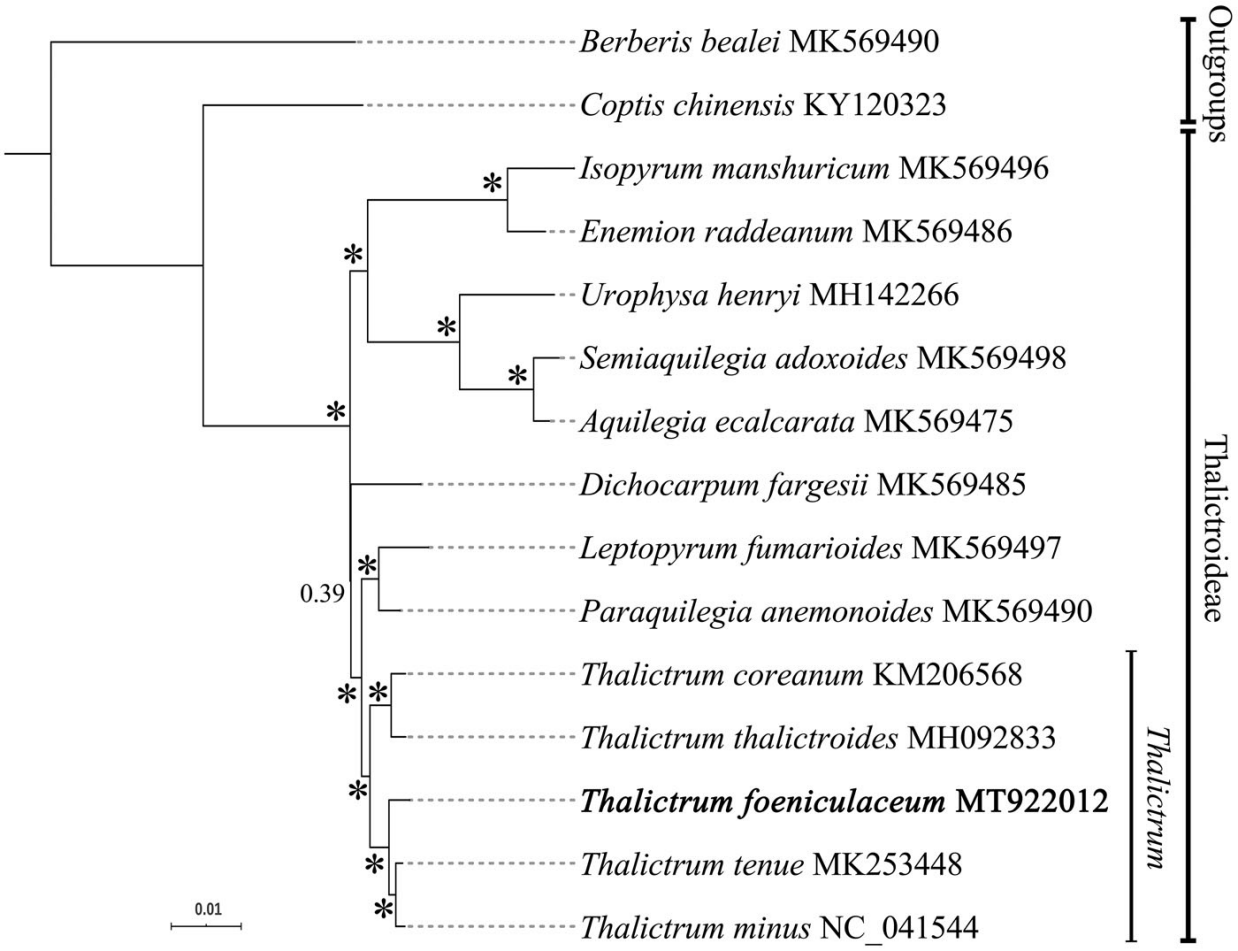
Bayesian phylogram of *Thalictrum* as well as its outgroups inferred from the complete chloroplast genome sequences. Internal branches which are fully supported by analyses (with 1 Bayesian values) were marked with *.

## Data Availability

The data that support the findings of this study are openly available in GenBank of NCBI at https://www.ncbi.nlm.nih.gov, reference number MT922012. Raw Illumina data is available at the Sequence Read Archive (SRA) under accession SRR12718335.
